# Real-Valued Covariance Vector Sparsity-Inducing DOA Estimation for Monostatic MIMO Radar

**DOI:** 10.3390/s151128271

**Published:** 2015-11-10

**Authors:** Xianpeng Wang, Wei Wang, Xin Li, Jing Liu

**Affiliations:** College of Automation, Harbin Engineering University, No. 145 Nantong Street, Harbin 150001, China; E-Mails: wangxianpeng@hrbeu.edu.cn (X.W.); xinxin-forever@sohu.com (X.L.); angleliujing@126.com (J.L.)

**Keywords:** MIMO radar, DOA estimation, sparse representation, unitary transformation, Khatri–Rao product

## Abstract

In this paper, a real-valued covariance vector sparsity-inducing method for direction of arrival (DOA) estimation is proposed in monostatic multiple-input multiple-output (MIMO) radar. Exploiting the special configuration of monostatic MIMO radar, low-dimensional real-valued received data can be obtained by using the reduced-dimensional transformation and unitary transformation technique. Then, based on the Khatri–Rao product, a real-valued sparse representation framework of the covariance vector is formulated to estimate DOA. Compared to the existing sparsity-inducing DOA estimation methods, the proposed method provides better angle estimation performance and lower computational complexity. Simulation results verify the effectiveness and advantage of the proposed method.

## 1. Introduction

Recently, multiple-input multiple-output (MIMO) radar has drawn considerable attention and become a hot research topic in the field of radar due to its advantages [1,2,3]. Based on the structure of transmit and receive arrays, MIMO radar can be divided into two classes: one is called statistical MIMO radar [2], and the other is called colocated MIMO radar [3] (including bistatic MIMO radar and monostatic MIMO radar). The transmit and receive arrays of the bistatic MIMO radar are separated from each other, so it can obtain the spatial gain from different direction channels. In colocated MIMO radar, the transmit and receive arrays are close to each other, which can improve the spatial resolution by forming a large aperture of the virtual array. In this paper, we focus on the monostatic MIMO radar, which is one kind of colocated MIMO radar.

Direction of arrival (DOA) estimation of multiple targets from the received data is an important aspect of array signal processing [4,5,6] and MIMO radar applications in practice [7,8,9,10,11]. Some subspace-based methods have been proposed for DOA estimation in MIMO radar, such as the multiple signal classification (MUSIC) algorithm [8], the estimation of signal parameters via rotational invariance techniques (ESPRIT) algorithm [9] and tensor analysis-based algorithms [10,11]. In addition, the reduced dimensional technique-based methods [12,13,14] are proposed for DOA estimation in monostatic MIMO radar by using the special structure of the virtual array. However, the above mentioned subspace-based methods have angle estimation performance degradation under lower SNR, closely spaced targets and limited snapshots. Recently, the sparse representation technique for signal recover has attracted much attention, and it has been applied to DOA estimation in array signal processing, such as $l_{1}$-norm singular value decomposition ($l_{1}$-SVD) [15], $l_{1}$-norm sparse representation of array covariance vectors ($l_{1}$-SRACV) [16], covariance matrix sparse representation (CMSR) [17] and the real-valued $l_{1}$-SVD method [18]. The simulation results have verified that compared to conventional subspace-based methods, these sparse representation-based methods have a better ability to adapt to many challenging scenarios, such as coherent targets, low SNR and limited snapshots. Furthermore, the $l_{1}$-norm-based sparse representation frameworks are proposed for angle estimation in MIMO radar [19,20]. However, they focus on the sparse representation of the received data in MIMO radar, which involves a high computational burden due to the multiple measurement vector (MMV) problem and complex-valued processing.

In this paper, we propose a real-valued covariance vector sparsity-inducing DOA estimation method for monostatic MIMO radar. The proposed method exploits the sparsity of the real-valued covariance vector for DOA estimation, which is different from the methods in [19,20]. In the proposed method, the reduced dimensional transformation and unitary transformation techniques are used to transform the received data into a low-dimensional real-valued one. Then, based on the Khatri–Rao product, a real-valued sparse representation framework of the covariance vector is formulated for DOA estimation when multiple snapshots are available. Due to the fact that the proposed method only involves real-valued processing and the single measurement vector (SMV) problem, it has lower computational complexity than existing sparsity-inducing DOA estimation methods. Furthermore, the proposed method has superior angle estimation performance when the number of snapshots is reasonable. The simulation results are used to verify the performance of the proposed method.

The rest of the paper is organized as follows. Section 2 introduces the monostatic MIMO radar signal model and the $l_{1}$-SVD algorithm for monostatic MIMO radar. Section 3 presents a real-valued sparse representation framework of the covariance vector for DOA estimation in monostatic MIMO radar. Section 4 presents some related remarks for this paper. Several numerical simulations are carried out in Section 5 to evaluate the performance of the proposed method. Finally, Section 6 concludes this paper.

Notation: ${\left(\cdot \right)}^{H}$, ${\left(\cdot \right)}^{T}$, ${\left(\cdot \right)}^{-1}$, ${\left(\cdot \right)}^{*}$ and ${\left(\cdot \right)}^{+}$ denote conjugate-transpose, transpose, inverse, conjugate and pseudo-inverse, respectively. ⊗ and ⊙ denote the Kronecker product operator and the Hadamard product operator, respectively. $I_{K}$ denotes a K×K-dimensional unit matrix, and E[·] denotes the expectation operator. $A^{\left(l_{2}\right)}$ denotes a column vector whose *q*-th element equals the $l_{2}$ norm of the *q*-th row of A. $\left|\right|\cdot {\left|\right|}_{1}$ and $\left|\right|\cdot {\left|\right|}_{F}$ denote the $l_{1}$ norm and the Frobenius norm, respectively.

## 2. The Monostatic MIMO Radar Signal Model and the $l_{1}$-SVD Algorithm

We consider a narrowband monostatic MIMO radar system equipped with *M* transmit antennas and *N* receive antennas. Both the transmit and receive arrays are half-wavelength-spaced uniform linear arrays (ULAs), and all of the elements are omnidirectional. At the transmit array, *M* transmit antennas emit *M* orthogonal narrow-band waveforms, which have identical bandwidth and center frequency. Let $g\left(t\right)\;=\;{\left[\phi_{1}\left(t\right),\cdots ,\phi_{M}\left(t\right)\right]}^{T}\in C^{M\times 1}$ be the orthogonal narrow-band transmitted waveforms, which satisfy:

(1) $$\int_{0}^{T_{q}}\phi_{i}\left(t\right)\phi_{j}^{*}\left(t\right)=\left\{\begin{array}{c} 1,i=j\\ 0,i\neq j \end{array}\right.$$

where $T_{q}$ is the pulse duration. It is assumed that there exists *P* uncorrelated targets located in the far-field region, and the DOA of the *p*-th target with respect to the transmit array and receive array is denoted as $\theta_{p}$. Then, the N×1 received data can be written as [7]:

(2) $$\bar{x}\left(t,\tau \right)=\sum_{p=1}^{P}\beta_{p}\left(\tau \right)e^{j2\pi f_{p}\left(\tau \right)}a_{r}\left(\theta_{p}\right)a_{t}^{T}\left(\theta_{p}\right)g\left(t\right)+\bar{n}\left(t,\tau \right)$$

where *τ* is the slow time index, *i.e.*, the pulse number (denoted as a snapshot in this paper), and $\beta_{p}\left(\tau \right)$ and $f_{p}\left(\tau \right)$ denote the reflection coefficient and Doppler frequency, respectively. $\bar{n}\left(t,\tau \right)\in C^{N\times M}$ is the noise matrix. The transmit steering vector $a_{t}\left(\theta_{p}\right)$ and the receive steering vector $a_{r}\left(\theta_{p}\right)$ are shown as:

$$\begin{array}{c} a_{t}\left(\theta_{p}\right)=\left\{\begin{array}{c} \begin{array}{c} {\left[e^{-j\frac{M-1}{2}\sin\theta_{p}},\cdots ,e^{-j\frac{1}{2}\sin\theta_{p}},e^{j\frac{1}{2}\sin\theta_{p}},\cdots ,e^{-j\frac{M-1}{2}\sin\theta_{p}}\right]}^{T},\;M\;\mathrm{is}\;\mathrm{even} \end{array}\\ {\left[e^{-j\frac{M-1}{2}\sin\theta_{p}},\cdots ,1,\cdots ,e^{-j\frac{M-1}{2}\sin\theta_{p}}\right]}^{T},\;M\;\mathrm{is}\;\mathrm{odd} \end{array}\right. \end{array}$$

and:

$$\begin{array}{c} a_{r}\left(\theta_{p}\right)=\left\{\begin{array}{c} \begin{array}{c} {\left[e^{-j\frac{N-1}{2}\sin\theta_{p}},\cdots ,e^{-j\frac{1}{2}\sin\theta_{p}},e^{j\frac{1}{2}\sin\theta_{p}},\cdots ,e^{-j\frac{N-1}{2}\sin\theta_{p}}\right]}^{T},\;N\;\mathrm{is}\;\mathrm{even} \end{array}\\ {\left[e^{-j\frac{N-1}{2}\sin\theta_{p}},\cdots ,1,\cdots ,e^{-j\frac{N-1}{2}\sin\theta_{p}}\right]}^{T},\;N\;\mathrm{is}\;\mathrm{odd} \end{array}\right. \end{array}$$

Exploiting the orthogonality property of the transmitted waveforms, the M×1 virtual data vectors can be obtained by matched filtering $\bar{x}\left(t,\tau \right)$ with each of the orthogonal waveforms $\phi_{m}\left(t\right),m=1,2,....,M$, which is given as follows:

(3) $$\begin{array}{cc} {\bar{x}}_{m}\left(\tau \right) & =\int_{0}^{T_{q}}\bar{x}\left(t,\tau \right)\phi_{m}^{*}\left(t\right)dt\\ & =\sum_{p=1}^{P}\beta_{p}\left(\tau \right)e^{j2\pi f_{p}\left(\tau \right)}a_{tm}\left(\theta_{p}\right)a_{r}\left(\theta_{p}\right)+{\bar{n}}_{m}\left(\tau \right) \end{array}$$

where $a_{tm}\left(\theta_{p}\right)$ is the *m*-th element of the transmit steering vector $a_{t}\left(\theta_{p}\right)$, and ${\bar{n}}_{m}\left(\tau \right)\;=\;\int_{0}^{T_{q}}\bar{n}\left(t,\tau \right)\phi_{m}^{*}\left(t\right)dt$ is the noise term. Then, stacking the individual vector components into one column vector, an MN×1 virtual vector can be obtained, which is written as [7,8,9]:

(4) $$\begin{array}{cc} x(\tau ) & ={\left[{\bar{x}}_{1}^{T}\left(\tau \right),{\bar{x}}_{2}^{T}\left(\tau \right),\cdots ,{\bar{x}}_{M}{\left(\tau \right)}^{T}\right]}^{T}\\ & =As(\tau )+n(\tau ) \end{array}$$

where $A=\left[a_{t}\left(\theta_{1}\right)⊗a_{r}\left(\theta_{1}\right),\cdots ,a_{t}\left(\theta_{P}\right)⊗a_{r}\left(\theta_{P}\right)\right]\in C^{NM\times P}$, $s\left(\tau \right)={\left[s_{1}\left(\tau \right),\cdots ,s_{P}\left(\tau \right)\right]}^{T}\in C^{P\times 1}$ is the signal vector with $s_{p}\left(\tau \right)=\beta_{p}\left(\tau \right)e^{j2\pi f_{p}\left(\tau \right)}$. $\bar{n}\left(\tau \right)={\left[n_{1}^{T}\left(\tau \right),\cdots ,{\bar{n}}_{M}^{T}\left(\tau \right)\right]}^{T}$ is the noise vector. When the number of snapshots is *J*, the Equation (4) can be expressed as:

(5) X=AS+N

where $X=\left[x\left(\tau_{1}\right),x\left(\tau_{2}\right)\cdots ,x\left(\tau_{J}\right)\right]\in C^{MN\times J}$ is the received data matrix. $S=[s\left(\tau_{1}\right),s\left(\tau_{2}\right)\cdots ,s\left(\tau_{J}\right)]$ and $N=[n\left(\tau_{1}\right),n\left(\tau_{2}\right),\cdots ,n\left(\tau_{J}\right)]$ are the signal data matrix and noise matrix, respectively. Some statistical assumptions on the target signals and noise are made as follows:

(i) The target signals are spatially uncorrelated, temporally white and zero-mean, *i.e.*, the Doppler frequencies $f_{p}\left(\tau \right)\left(p=1,2,...,P\right)$ are different, and the reflection coefficients $\beta_{p}\left(\tau_{k}\right)\;\left(k\;=\;1,2,...,J\right)$ obey the Swerling II model.
(ii) The noise matrix N is zero-mean, complex circular Gaussian and with the variance $R_{n}=\delta^{2}I_{MN}$.
(iii) The noise is statistically independent of all targets.

Based on the above statistical assumptions, the work is to estimate the DOA from the received data X in this paper. Firstly, we introduce the $l_{1}$-SVD algorithm for DOA estimation. Due to the fact that the DOAs of targets are sparse when the discretized sampling grid number of all spatial spaces is much larger than the target number, the $l_{1}$-SVD algorithm [14] can be applied to DOA estimation in monostatic MIMO radar. After using the SVD technique, the received data can be written as:

(6) $$X_{SV}=AS_{SV}+N_{SV}$$

where $X_{SV}=XV_{s}$, $S_{SV}=SV_{s}$ and $N_{SV}=NV_{s}$. $V_{s}\in C^{J\times P}$ is composed with the singular vectors corresponding to the *P* largest singular values of X. Then, Equation (6) can be sparsely represented by using the sampling grid $\Theta =[{\hat{\theta }}_{1},{\hat{\theta }}_{2},\cdots ,{\hat{\theta }}_{L}]$, where L≥P. The transmit and receive complete dictionaries are expressed as $A_{t}^{\hat{\theta }}=\left[a_{t}\left({\hat{\theta }}_{1}\right),\cdots ,a_{t}\left({\hat{\theta }}_{L}\right)\right]$ and $A_{r}^{\hat{\theta }}=\left[a_{r}\left({\hat{\theta }}_{1}\right),\cdots ,a_{t}\left({\hat{\theta }}_{L}\right)\right]$, respectively. Thus, the complete dictionary can be constructed as $A_{\hat{\theta }}=A_{t}^{\hat{\theta }}⊙A_{r}^{\hat{\theta }}$. Then, under the framework of the sparse representation, Equation (6) can be formulated as:

(7) $$X_{SV}=A_{\hat{\theta }}S_{SV}^{\hat{\theta }}+N_{SV}$$

where $S_{SV}^{\hat{\theta }}\in C^{L\times P}$ and $S_{SV}$ have the same row support, *i.e.*, the matrix $S_{SV}^{\hat{\theta }}$ is sparse. In order to estimate $S_{SV}^{\hat{\theta }}$, Equation (7) can be formulated as the $l_{1}$-norm minimization problem, which is shown as follows:

(8) $$\min∥{\left(S_{SV}^{\hat{\theta }}\right)}^{\left(l_{2}\right)}{∥}_{1},\;s.t.∥X_{SV}-A_{\hat{\theta }}S_{SV}^{\hat{\theta }}{∥}_{2}\leq \bar{\eta }$$

where $\bar{\eta }$ is the regularization parameter. After obtaining the sparse matrix $S_{SV}^{\hat{\theta }}$, the DOAs can be estimated by finding non-zero rows of $S_{SV}^{\hat{\theta }}$. According to Equation (8), the procedure of recovering $S_{SV}^{\hat{\theta }}$ involves the MMV problem and complex-valued processing. Thus, the $l_{1}$-norm-based sparse representation methods lead to high computational complexity. On the other hand, the spatial resolution depends on the aperture of the MIMO radar in Equation (8), which has limited ability to estimate the DOA when the targets are located closely. In the next section, we propose a real-valued sparse representation framework of the covariance vector for DOA estimation to solve the drawbacks mentioned above.

## 3. Real-Valued Sparse Representation Framework of the Covariance Vector for DOA Estimation

When applying the $l_{1}$-norm-based sparse representation methods to estimate the DOA in MIMO radar, the MMV problem and complex-value processing are involved [19,20]. Thus, the computational complexity can be significantly reduced if the MMV problem can be converted into the SMV problem, and only real-valued processing is required in the $l_{1}$-norm minimization problem. In addition, the aperture of MIMO radar can be enlarged if the covariance matrix can be vectorized. These motivate us to propose a real-valued sparse representation framework of the covariance vector for DOA estimation in monostatic MIMO radar.

### 3.1. Reduced Dimension and Real-Value Transformation

According to the special configuration of monostatic MIMO radar, there are only Q=M+N-1 distinct elements. Therefore, the steering vector $a_{t}\left(\theta \right)⊗a_{r}\left(\theta \right)$ can be expressed as:

(9) $$a_{t}\left(\theta \right)⊗a_{r}\left(\theta \right)=G_{MN\times Q}b\left(\theta \right)$$

where $G_{MN\times Q}$ and b(θ) are the reduced dimensional transformation matrix and the steering vector, respectively, and they can be written as:

(10) $$G_{MN\times Q}={\left[J_{0}^{T},J_{1}^{T},\cdots ,J_{M-1}^{T}\right]}^{T}$$

(11) $$\begin{array}{c} b\left(\theta \right)=\left\{\begin{array}{c} \begin{array}{c} {\left[e^{-j\frac{Q-1}{2}\sin\theta },\cdots ,e^{-j\frac{1}{2}\sin\theta },e^{j\frac{1}{2}\sin\theta },\cdots ,e^{-j\frac{Q-1}{2}\sin\theta_{p}}\right]}^{T},\;Q\;\mathrm{is}\;\mathrm{even} \end{array}\\ {\left[e^{-j\frac{Q-1}{2}\sin\theta_{p}},\cdots ,1,\cdots ,e^{-j\frac{Q-1}{2}\sin\theta_{p}}\right]}^{T},\;Q\;\mathrm{is}\;\mathrm{odd} \end{array}\right. \end{array}$$

where $J_{m}=\left[0_{N\times m},I_{N},0_{N\times (M-m-1)}\right],\;m=0,1,...,M-1$. Based on Equation (10), we define a matrix as $F=G^{H}G$, which can be written as:

(12) $$F=\mathrm{diag}[1,2,...,\underset{|M-N|+1}{\underset{︸}{\min(M,N),...,\min(M,N)}},....,2,1]$$

In order to hold the characteristic of the noise matrix N, the reduce-dimensional matrix is defined as $W=F^{-(1/2)}G^{H}$[14], which satisfies $WW^{H}=I_{M+N-1}$. Then, multiplying the received signal X by W, we have:

(13) $$\begin{array}{c} Y=F^{\left(1/2\right)}BS+WN=\hat{B}S+WN \end{array}$$

where $B=\left[b\left(\theta_{1}\right),b\left(\theta_{2}\right),...,b\left(\theta_{P}\right)\right]\in C^{Q\times P}$ and $\hat{B}=F^{\left(1/2\right)}B$. Then, the received data in Equation (13) correspond to the effective steering vector b(θ), which can be used for DOA estimation. The sparse representation framework for DOA estimation in [19] is based on received data, and the $l_{1}$-norm minimization problem involves the MMV problem and complex-valued processing. In order to avoid this issue, a real-valued sparse representation framework of the covariance vector with the SMV problem is proposed as follows. Based on Equation (13), we note that the steering matrix becomes $\hat{B}$, which satisfies:

(14) $$\Pi_{M+N-1}{\hat{B}}^{*}=\hat{B}$$

where $\Pi_{K}$ denotes the K×K exchange matrix with ones on its anti-diagonal and zeros elsewhere. It can be concluded from Equation (14) that the linear array is a centro-symmetric array after the reduced-dimensional transformation. Therefore, the unitary transformation can be adopted to convert the complex-valued steering matrix into a real-valued one. Following the convention in [21], the unitary transformation matrix is defined as follows:

(15) $$U_{2K}=\frac{1}{\sqrt{2}}\left[\begin{array}{cc} I_{K} & jI_{K}\\ \Pi_{K} & -j\Pi_{K} \end{array}\right]$$

and:

(16) $$U_{2K+1}=\frac{1}{\sqrt{2}}\left[\begin{array}{ccc} I_{K} & 0 & jI_{K}\\ 0^{T} & \sqrt{2} & 0^{T}\\ \Pi_{K} & 0 & -j\Pi_{K} \end{array}\right]$$

Then, multiplying the received data Y by the unitary transformation matrix $U_{Q}$, a new received data matrix can be expressed as:

(17) $$\hat{Y}=U_{Q}^{H}Y=U_{Q}^{H}\hat{B}S+U_{Q}^{H}WN$$

According to Equation (17), after the unitary transformation, the complex-valued steering matrix is converted into real-valued steering matrix $\bar{B}=U_{Q}^{H}\hat{B}$. Then, the data matrix $\bar{Y}$ can be divided into real and imaginary parts, respectively, which are shown as:

(18) Re(Y^)=B¯Re(S)+Re(UQHWN)

(19) Im(Y^)=B¯Im(S)+Im(UQHWN)

where Re(·) and Im(·) denote the real part and the imaginary part, respectively. Using Equations (18) and (19), a real-valued augmentation data matrix can be formulated, which is expressed as:

(20) Y¯=[Re(Y^),Im(Y^)]=B¯S¯+N¯

where S¯=[Re(S),Im(S)]∈RP×2J and N¯=[Re(UQHWN),Im(UQHWN)]∈RQ×2J. With the statistical Assumptions (i), (ii) and (iii), the covariance matrix of $\bar{S}$ is $R_{S}=E\left[\bar{S}{\bar{S}}^{H}\right]=\mathrm{diag}\left(d\right)$ with diagonal elements $d={\left[d_{1}^{2},....,d_{P}^{2}\right]}^{T}$ being the signal power vector.

### 3.2. Real-Valued Sparse Representation Framework for DOA Estimation

According to Equation (20), the real-valued covariance matrix of $\bar{Y}$ can be expressed as:

(21) $$\begin{array}{cc} R_{\bar{Y}} & =E\left[\bar{Y}{\bar{Y}}^{H}\right]=\bar{B}R_{S}{\bar{B}}^{H}+\sigma^{2}I_{Q}\\ & =\bar{B}\mathrm{diag}\left(d\right){\bar{B}}^{H}+\sigma^{2}I_{Q} \end{array}$$

Based on the Khatri–Rao product, the vectorization of the covariance matrix provides a new way for array augmentation [22]. Thus, exploiting the vectorization (vec) operator on $R_{\bar{Y}}$, we have:

(22) $$\begin{array}{cc} y & =\mathrm{vec}\left(R_{\bar{Y}}\right)=\left(\bar{B}⊙\bar{B}\right)d+\sigma^{2}\mathrm{vec}\left(I_{Q}\right) \end{array}$$

According to Equation (22), the data vector y can be considered as a new signal model corresponding to a virtual array output with a single snapshot, and the effective array response vector of the virtual array can be regarded as b(θ)⊗b(θ). According to the received data in Equation (5), the effective array response vector of the received data is b(θ). Thus, the proposed signal model in Equation (22) significantly increases the circs of freedom (DOF) of the virtual array and shows an enlarged aperture. Moreover, the sparse representation framework for Equation (22) is only involved with the SMV problem, which can remarkably reduce the computational complexity compared to the MMV problem in Equation (5).

Based on the proposed signal model in Equation (22), a real-valued sparse representation scheme of the covariance vector for DOA estimation is formulated. Let ${\hat{\theta }}_{1},{\hat{\theta }}_{2},\cdots ,{\hat{\theta }}_{L}$ be the discretized sampling grid of the spatial domain of interest. The number of potential DOAs will be much greater than the number of targets, *i.e.*, L≫P. Then, the one-dimensional complete dictionary for DOA estimation can be constructed as ${\underline{B}}_{\hat{\theta }}={\bar{B}}_{\hat{\theta }}⊙{\bar{B}}_{\hat{\theta }}$, where ${\bar{B}}_{\hat{\theta }}=U_{Q}^{H}F^{\left(1/2\right)}B_{\hat{\theta }}$ with $B_{\hat{\theta }}=\left[b\left({\hat{\theta }}_{1}\right),...,b\left({\hat{\theta }}_{L}\right)\right]$. Under the sparse representation framework, Equation (22) can be represented with the complete dictionary as:

(23) $$y={\underline{B}}_{\hat{\theta }}d_{\hat{\theta }}+\sigma^{2}\mathrm{vec}\left(I_{Q}\right)$$

where $d_{\hat{\theta }}\in C^{L\times 1}$ is a *P*-sparse vector, which has *P* non-zero elements. Thus, the DOAs can be estimated by detecting the non-zeros elements of $d_{\hat{\theta }}$.

In practice, the unknown covariance matrix can be estimated by ${\hat{R}}_{\bar{Y}}=\left(1/J\right)\bar{Y}{\bar{Y}}^{H}$. Then, we have $\hat{y}=\mathrm{vec}\left({\hat{R}}_{\bar{Y}}\right)$ and $\hat{y}-y=▵y$, where ▵y is the estimation error. Then, combining the sparse representation framework in Equation (23) and the estimation error ▵y, a real-valued $l_{1}$ norm minimization problem with SMV is formulated as:

(24) $$\min∥d_{\hat{\theta }}{∥}_{1}\;s.t.∥\hat{y}-{\underline{B}}_{\hat{\theta }}d_{\hat{\theta }}-\sigma^{2}\mathrm{vec}\left(I_{Q}\right){∥}_{2}^{2}\leq \eta$$

where *η* is a regularization parameter, which sets the amount of error and plays an important role in the final DOA estimation performance. According to the $l_{1}$ norm minimization problem in Equation (24), the selection of parameter *η* depends on the distribution of ▵y.

Lemma 1: After using the reduced dimensional transformation and unitary transformation, the estimation error ▵y satisfies $▵y∼AsN(0,\frac{1}{J}R_{\bar{Y}}^{T}⊗R_{\bar{Y}})$, where $AsN(\mu ,\sigma^{2})$ represents the asymptotic normal distribution with mean *μ* and variance $\sigma^{2}$.

Proof: According to the definition of W and $U_{Q}$, both of them are orthogonal matrices. Thus, ${\left[U_{Q}^{H}\hat{B}S\right]}_{i,j}$ and ${\left[U_{Q}^{H}WN\right]}_{i,j}$ have a complex Gaussian distribution with zero mean, because the orthogonal invariance property of the Gaussian random matrix makes the distribution impervious to multiplication by orthogonal matrices, where ${\left[\cdot \right]}_{i,j}$ denotes the (i,j)-th element of a matrix. Furthermore, due to the fact that the real part and imaginary part of ${\left[U_{Q}^{H}\hat{B}S\right]}_{i,j}$ and ${\left[U_{Q}^{H}WN\right]}_{i,j}$ are real a Gaussian distribution with zero mean, ${\left[\bar{B}\bar{S}\right]}_{i,j}$ and ${\left[\bar{N}\right]}_{i,j}$ satisfy the Gaussian distribution with zero mean. Thus, according to [23], it can be concluded that the estimation error $▵y=\hat{y}-y$ satisfies:

(25) $$▵y=\mathrm{vec}\left({\hat{R}}_{\bar{Y}}-R_{\bar{Y}}\right)∼AsN\left(0,\frac{1}{J}R_{\bar{Y}}^{T}⊗R_{\bar{Y}}\right)$$

According to Equation (25), it is noticed that ▵y is not the asymptotic standard normal distribution, and the parameter *η* cannot be calculated in an easy way. However, it is obvious that a weighted matrix ${\bar{W}}^{-\frac{1}{2}}=\frac{1}{J}R_{\bar{Y}}^{-\frac{T}{2}}⊗R_{\bar{Y}}^{-\frac{1}{2}}$ can be formulated to make the estimation error ▵y satisfy the asymptotic standard normal distribution; then, we further have:

(26) $$∥{\bar{W}}^{-\frac{1}{2}}▵y{∥}_{2}^{2}∼As\chi^{2}\left(0,I_{Q^{2}}\right)$$

where $As\chi^{2}\left(0,I_{Q^{2}}\right)$ denotes the asymptotic chi-square distribution with $Q^{2}$ circs of freedom. Combining Equation (24) and Equation (26), the weighted real-valued $l_{1}$ norm minimization problem with SMV is formulated as:

(27) $$\begin{array}{cc} & \;\;\;\;\;\;\min∥d_{\hat{\theta }}{∥}_{1}\\ & s.t.∥{\hat{W}}^{-\frac{1}{2}}\left(\hat{y}-{\hat{\sigma }}^{2}\mathrm{vec}\left(I_{Q}\right)\right)-\left({\hat{W}}^{-\frac{1}{2}}{\underline{B}}_{\hat{\theta }}\right)d_{\hat{\theta }}{∥}_{2}\leq \sqrt{\eta_{1}} \end{array}$$

where ${\hat{W}}^{-\frac{1}{2}}$ is the estimation of ${\bar{W}}^{-\frac{1}{2}}$ and can be calculated as ${\hat{W}}^{-\frac{1}{2}}=\frac{1}{J}{\hat{R}}_{\bar{Y}}^{-\frac{T}{2}}⊗{\hat{R}}_{\bar{Y}}^{-\frac{1}{2}}$. According to Equation (26), the parameter $\eta_{1}$ can be selected with a high probability 1-ξ, where *ξ* is a small value. Usually, it is enough to set ξ=0.001 for calculating the value of $\eta_{1}$, which can be implemented via the function $\eta_{1}=chi2inv\left(1-\xi ,Q^{2}\right)$ in MATLAB software. ${\hat{\sigma }}_{r}^{2}$ is the estimation of ${\hat{\sigma }}_{r}^{2}$ and can be estimated by the average of the Q-P smallest eigenvalue or the minimum eigenvalue of ${\hat{R}}_{\bar{Y}}$. Equation (27) can be calculated by SOC (second order cone) programming software packages, such as Sedumi [24] and CVX [25]. Then, a spatial spectrum can be obtained by plotting $d_{\hat{\theta }}$ solved from Equation (27), and the DOA can be estimated by finding the *P* largest values.

## 4. Related Remarks

Remark 1: In order to obtain the computational complexity of the sparsity-inducing DOA estimation methods, we firstly analyze the computational complexity of solving the SOC programming, such as in Equations (8) and (27). Referring to the conclusion in [15], for the observing matrix $X_{SV}\in C^{MN\times P}$, the computational complexity of solving SOC programming in Equation (8) is $O(L^{3}P^{3})$ flops when L≫MN, where flops is the abbreviation of floating-point operations. For the the observing vector $\hat{y}\in C^{Q^{2}\times 1}$ in the proposed method, the computational complexity of solving the SOC programming in Equation (27) is $O(L^{3})$ due to $L\gg Q^{2}$. Thus, the computational complexity analysis of the proposed method is given as follows. As shown in the steps of the proposed method, the main computational complexity of the proposed method contains three parts: (1) the reduced dimensional transformation in Equation (13) requires O(QMNJ) flops; (2) the unitary transformation in Equation (17) and calculating real-valued covariance matrix in Equation (21) require $O(2Q^{2}J)$ flops; (3) solving the SOC programming in Equation (27) with real-valued processing requires $O(\frac{1}{4}L^{3})$ flops. Thus, the computational complexity of the proposed method is $O(QMNJ+2Q^{2}J+\frac{1}{4}L^{3})$ flops. For the complex-valued $l_{1}$-SVD algorithm [15] with the received data in MIMO radar, the computational complexity focus on the SVD of the received data in Equation (5) and solving the SOC programming in Equation (8), which requires $O(M^{3}N^{3}+L^{3}P^{3})$ flops. For the real-valued (RV) $l_{1}$-SVD algorithm, it only requires the additions to transform the complex received data into a real one compared to the $l_{1}$-SVD algorithm. Thus, it requires $O(MNJ+\frac{1}{4}M^{3}N^{3}+\frac{1}{4}L^{3}P^{3})$. Owing to L≫M,N,J,Q, the proposed method has lower computational complexity than the RV $l_{1}$-SVD and $l_{1}$-SVD algorithms.

Remark 2: Based on the principle of the proposed method mentioned above, it can be concluded that the proposed method has better angle estimation performance and higher angular resolution than conventional $l_{1}$-norm based algorithms when the covariance matrix can be estimated efficiently, *i.e*, the number of snapshots is reasonable. This is because exploiting the covariance matrix vectorization technique in Equation (22), the virtual aperture corresponding to y has been enlarged remarkably, *i.e.*, the angular resolution can be improved.

Remark 3: The maximal number of identified targets is an important aspect that can be considered for the sparse representation-based algorithms. Then, according to [21], any set of 2Q-1 columns of the complete dictionary ${\underline{B}}_{\hat{\theta }}$ is independent, which leads to $\mathrm{Spark}[{\underline{B}}_{\hat{\theta }}]=2Q$, where $\mathrm{Spark}[\Omega_{\bar{\theta }}]$ denotes the smallest integer of columns of the complete dictionary ${\underline{B}}_{\hat{\theta }}$ that are linearly dependent. Then, the maximal number of identified targets is $\mathrm{Spark}[{\underline{B}}_{\hat{\theta }}]/2-1=Q-1$ in the proposed method [26]. Thus, though the virtual aperture is enlarged, the proposed method cannot handle the underdetermined DOA estimation case.

Remark 4: According to Equation (22), if the target signals are coherent, the signal power vector d exists with zero elements, and the vector y is meaningless. Thus, the proposed method is not suitable for the coherent targets. Additionally, as shown in Equations (21) and (22), the proposed method is based on the assumption that $R_{S}$ is a diagonal matrix. The matrix $R_{S}$, in fact, does not satisfy the diagonal matrix when the number of snapshots is limited. Therefore, the angle estimation performance of the proposed method degrades remarkably with lower snapshots.

## 5. Simulation Results

In this section, we carry out some simulations to evaluate the proposed method. We also compare the proposed methods with the $l_{1}$-SVD algorithm [15], the real-valued $l_{1}$-SVD algorithm (denoted as RV $l_{1}$-SVD) [18] and the Cram$\overset{´}{e}$r–Rao bound (CRB) [27]. In most of the simulations, a monostatic MIMO radar with M=N=5 colocated antennas for the transmit and receive arrays is used. Both of them are half-wavelength-spaced ULAs. The signal-to-noise ratio (SNR) is defined as 10log$\left(\sigma_{s}^{2}/\sigma_{n}^{2}\right)$, where $\sigma_{s}^{2}$ and $\sigma_{n}^{2}$ denote the signal and noise power, respectively. The root-mean-square-error (RMSE) of the DOA estimation obtained from 300 Monte Carlo runs is defined as:

(28) $$\begin{array}{c} \mathrm{RMSE}=\sqrt{\frac{1}{300P}\sum_{i=1}^{300}\sum_{p=1}^{P}{\left({\hat{\theta }}_{i,p}-\theta_{p}\right)}^{2}} \end{array}$$

where ${\hat{\theta }}_{i,p}$ denotes the estimation of $\theta_{p}$ at the *i*-th trial. For all methods in the simulations, the number of targets is assumed to be known or estimated by the minimum description length (MDL) criterion, and the confidence interval is set to 0.999 for the $l_{1}$-SVD algorithm, the RV $l_{1}$-SVD algorithm and the proposed method (*i.e.*, ξ=0.001). The spatial grid is uniform from the range $-90^{\circ }$ to $90^{\circ }$ with $0.1^{\circ }$ interval in all simulations.

**Figure 1:**
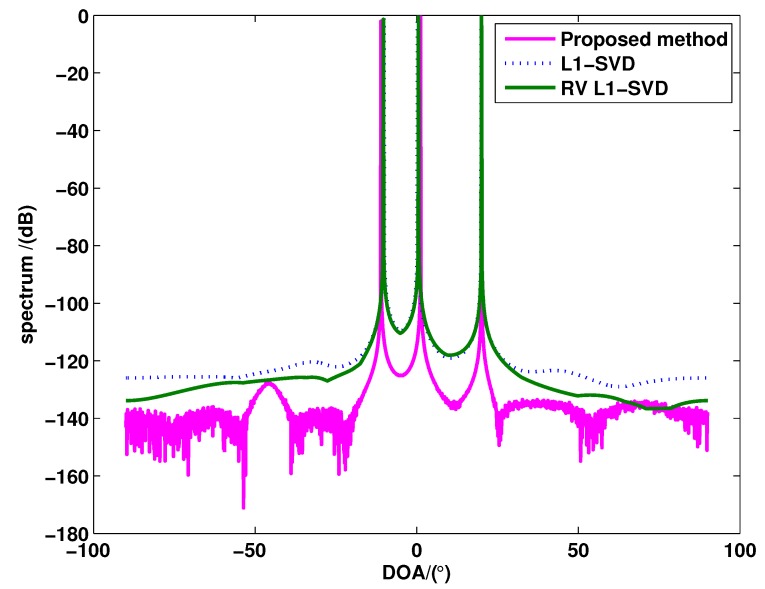
The spectra of $l_{1}$-SVD, real-valued (RV) $l_{1}$-SVD and the proposed method for uncorrelated targets.

Figure 1 shows the spectra of these three algorithm, where P=3 uncorrelated targets are assumed to be with the DOAs as $\theta_{1}=-10.13^{\circ }$, $\theta_{2}=0.13^{\circ }$ and $\theta_{3}=20.13^{\circ }$. The SNR and snapshots are fixed with 0 dB and 400, respectively. As can be seen from Figure 1, compared to the RV $l_{1}$-SVD algorithm and the $l_{1}$-SVD algorithm, the proposed method has a lower sidelobe, which means that the proposed method has higher angular resolution.

**Figure 2:**
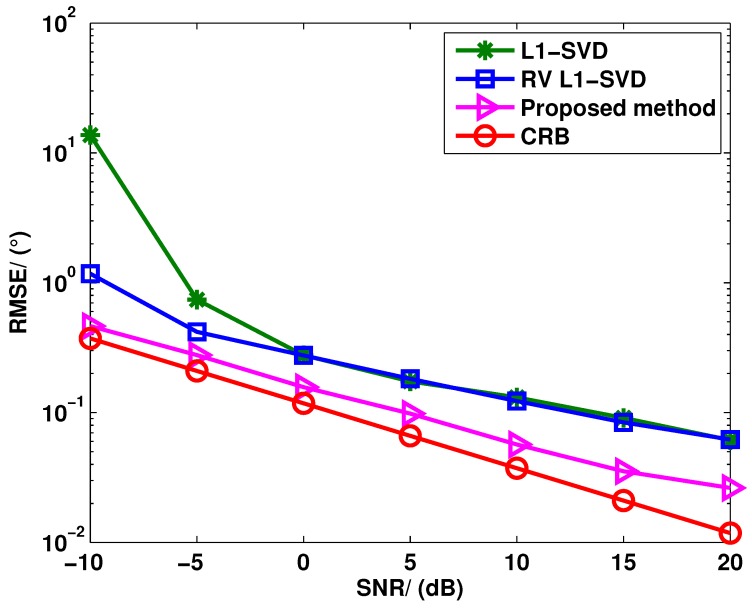
RMSE of $l_{1}$-SVD, RV $l_{1}$-SVD and the proposed method for uncorrelated targets when the SNR varies from −10 dB to 20 dB.

**Figure 3:**
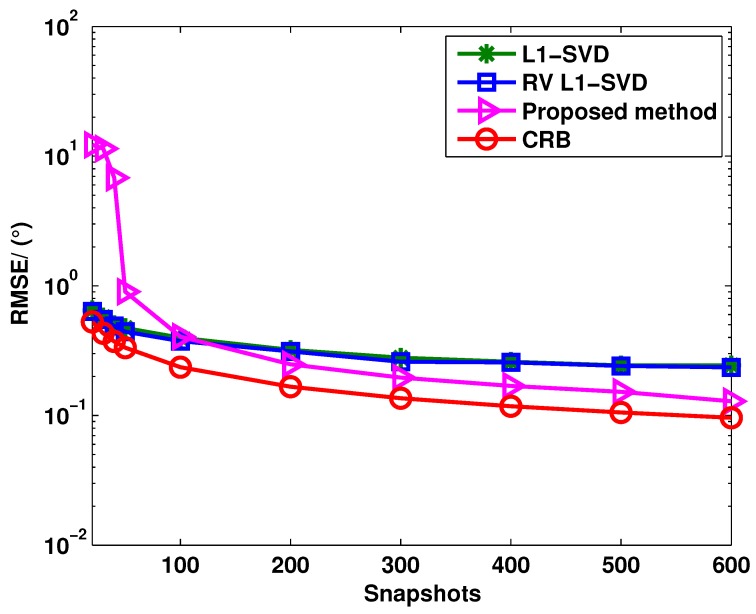
RMSE of $l_{1}$-SVD, RV $l_{1}$-SVD and the proposed method for uncorrelated targets when the snapshots number varies from 20 to 600.

Figure 2 and Figure 3 show the angle estimation performance of these three algorithms *versus* the SNR and snapshots, respectively, where P=3 uncorrelated targets are assumed to be with the DOAs as $\theta_{1}=-10.13^{\circ }$, $\theta_{2}=0.13^{\circ }$ and $\theta_{3}=20.13^{\circ }$. In Figure 2, we keep the snapshots fixed at 400, and the SNR is varied from −10 dB to 20 dB; in Figure 3, the SNR is fixed at 0 dB, and the number of snapshots is varied from 50 to 600. It can be seen from Figure 2 that the RV $l_{1}$-SVD algorithm provides better angle estimation performance than the $l_{1}$-SVD algorithm at the low SNR region and comparable angle estimation performance at the high SNR region. Compared to the RV $l_{1}$-SVD and $l_{1}$-SVD algorithms, the proposed method outperforms both of them at all SNR regions. The reason is that the virtual aperture is enlarged in the proposed method. From Figure 3, it can be concluded that the proposed method provides better angle estimation performance than both the RV $l_{1}$-SVD and $l_{1}$-SVD algorithms when the number of snapshots satisfies J>100. However, the angle estimation performance is deteriorated in the adaptation to much lower snapshots. This is because the covariance vectors become heavily perturbed when the snapshot number is small. Thus, the proposed method provides better angle estimation performance with a sufficiently large sample size.

**Figure 4:**
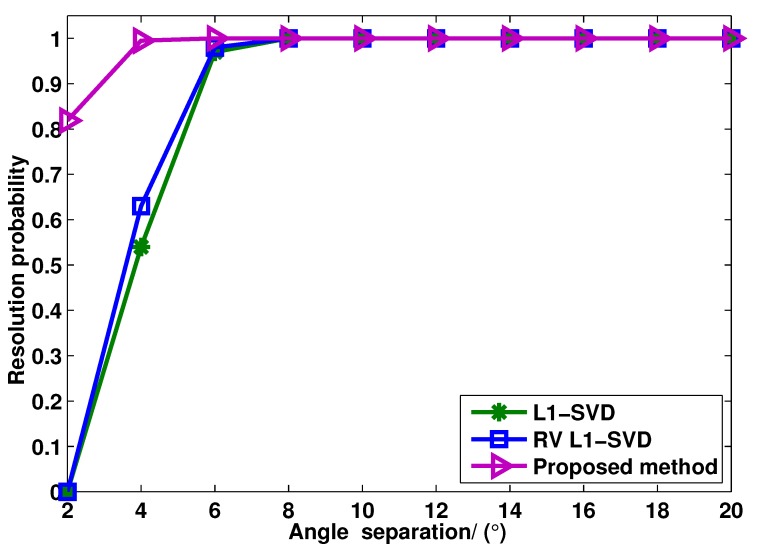
Target resolution probability of $l_{1}$-SVD, RV $l_{1}$-SVD and the proposed method for two uncorrelated targets when the angle separation varies from $2^{\circ }$ to $20^{\circ }$.

Figure 4 shows the target resolution probability of these three algorithms *versus* angle separation, where the SNR and snapshot number are fixed at 0 dB and 400, respectively. Two uncorrelated targets are considered with the DOAs as $\theta_{1}=0.13^{\circ }$ and $\theta_{2}=0.13^{\circ }+▵\theta$, where ▵θ varies from $2^{\circ }$ to $20^{\circ }$. In this simulation, two targets can be considered to be resolved if there are at least two peaks in the spatial spectrum, and it satisfies $max_{i=1,2}|{\hat{\theta }}_{i}-\theta_{i}|\leq \bar{▵}\theta /2$, where $\bar{▵}\theta /2=\left|\theta_{2}-\theta_{1}\right|$ and ${\hat{\theta }}_{i}$ is the estimation of $\theta_{i}$. As can be seen from Figure 4, compared to the RV $l_{1}$-SVD and $l_{1}$-SVD algorithms, the proposed method shows the highest target resolution probability for closely-spaced targets. The reason is that the virtual aperture is remarkably enlarged in the proposed method, *i.e.*, the angular resolution is improved.

**Figure 5:**
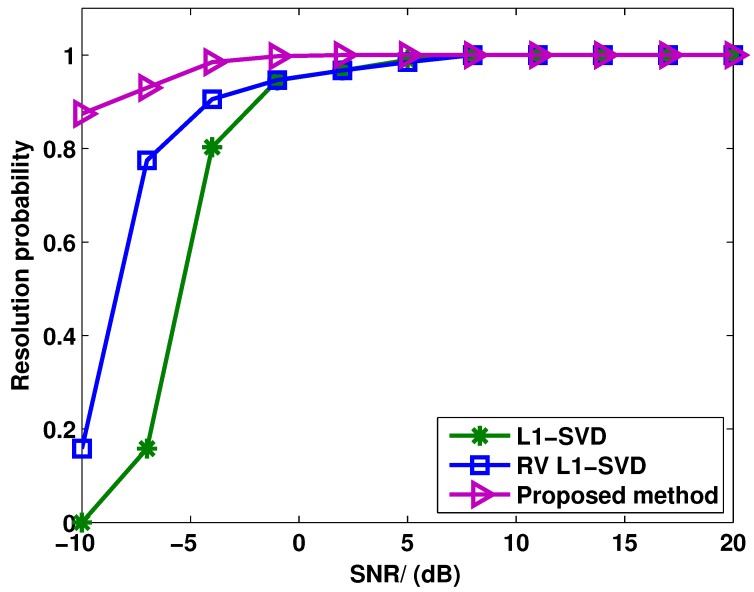
Target resolution probability of $l_{1}$-SVD, RV $l_{1}$-SVD and the proposed method for uncorrelated targets when the SNR varies from −10 dB to 20 dB.

Figure 5 shows the target resolution probability of these three algorithms *versus* SNR, where the snapshots number is fixed at 400. Two uncorrelated closely-spaced targets are considered with the DOAs as $\theta_{1}=0.52^{\circ }$ and $\theta_{2}=4.52^{\circ }$. The definition of target resolution is the same as Figure 4. It can be seen from Figure 5 that all methods exhibit a 100% correct target resolution at the high SNR region. As the SNR decreases, the probability of target resolution starts dropping for each method at a certain point, which is known as the SNR threshold. From Figure 5, it can be seen that the proposed method has a lower SNR threshold compared to the RV $l_{1}$-SVD and $l_{1}$-SVD algorithms, *i.e.*, the proposed method shows the best capability in resolving closely-spaced targets.

**Figure 6:**
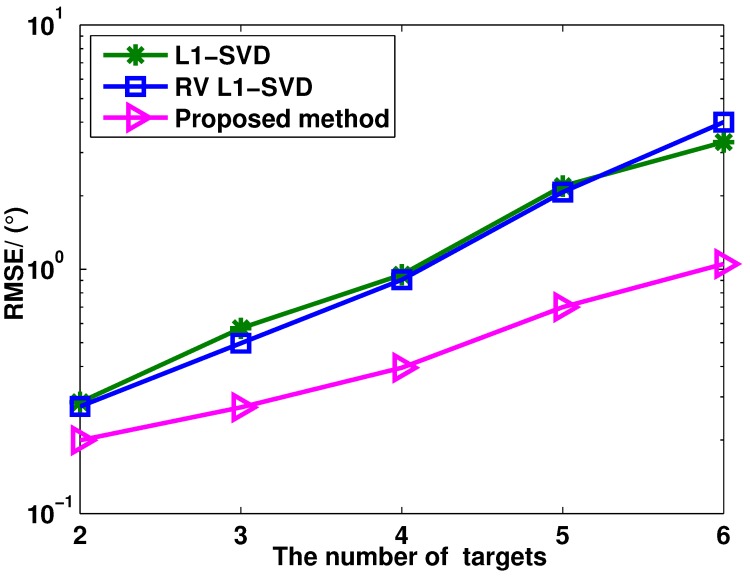
RMSE of $l_{1}$-SVD, RV $l_{1}$-SVD and the proposed method *versus* the number of targets.

**Figure 7:**
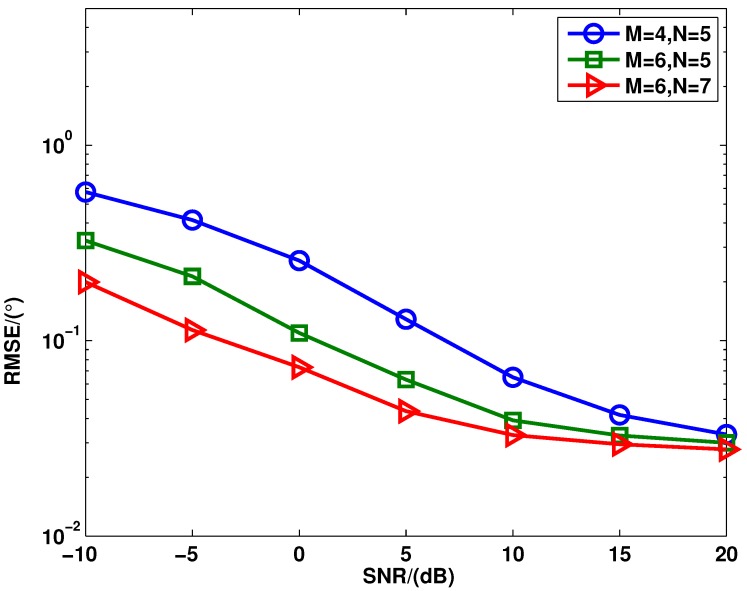
RMSE of the proposed method *versus* different elements when the SNR varies from −10 dB to 20 dB.

Figure 6 shows the RMSE of these three algorithms *versus* the number of targets, where the SNR = 0 dB and the number of snapshots is fixed at 400. The DOA of the *p*th target is $-30^{\circ }+\left(p-1\right)10^{\circ }$. From Figure 6, it can be seen that the proposed method provides better angle estimation performance than the RV $l_{1}$-SVD and $l_{1}$-SVD algorithms with different numbers of targets. This is because the proposed method can enlarge the aperture of the virtual array. Figure 7 shows the RMSE of the proposed method *versus* different transmit and receive elements, where P=3 uncorrelated targets are assumed to be with the DOAs as $\theta_{1}=-10.13^{\circ }$, $\theta_{2}=0.13^{\circ }$ and $\theta_{3}=20.13^{\circ }$, and the number of the snapshots is fixed as 400. As shown in Figure 7, the proposed method has improved angle estimation performance when the number of elements in the transmit or receive array increases. This is because of the diversity gain.

The angle estimation performance of the proposed method has been evaluated with the foregoing simulations. Here, the computational complexity of $l_{1}$-SVD, RV $l_{1}$-SVD and the proposed method is evaluated by using the time in count (TIC) and time out count (TOC) instruction in MATLAB software. The number of targets *P* is assumed to be known beforehand. The SNRs of all targets are set at 10 dB, and the number of snapshots is 400. The DOAs of targets satisfy $\theta_{i+1}-\theta_{i}=10^{\circ }\left(i=1,2,...,P-1\right)$. For each target number, the computation time of these algorithms is averaged over 300 trials. The computation time comparison of these algorithms is shown in Table 1. As can be seen from Table 1, the computation time of the $l_{1}$-SVD and RV $l_{1}$-SVD algorithms increases markedly when the number of targets increases, but the computation time of the proposed method is similar. On the other hand, the computation time of the proposed method is lower than both the $l_{1}$-SVD and RV $l_{1}$-SVD algorithms.

**Table 1:** Computation time comparison of $l_{1}$-SVD, RV $l_{1}$-SVD and the proposed method for uncorrelated targets when the targets number varies from 3 to 6.

Target Number	Average Computation Time (s)		
	$l_{1}$-SVD	RV $l_{1}$-SVD	Proposed Method
P = 3	3.7392	3.2885	2.4312
P = 4	7.8878	4.1707	2.5367
P = 5	9.6259	5.4507	2.7102
P = 6	11.1161	5.9464	2.7992

## 6. Conclusions

In this paper, we proposed a real-valued sparse representation framework of covariance vector for DOA estimation in MIMO radar. The proposed method exploits the reduced dimension and unitary transformation technique to turn the received data into a low dimensional real-valued one, then a sparse representation framework of the covariance vector is formulated for DOA estimation. Both the theoretical analysis and simulation results verify that the proposed method provides better angle estimation performance and has lower computational complexity than both the $l_{1}$-SVD and RV $l_{1}$-SVD algorithms.
